# High Fructose Corn Syrup-Moderate Fat Diet Potentiates Anxio-Depressive Behavior and Alters Ventral Striatal Neuronal Signaling

**DOI:** 10.3389/fnins.2021.669410

**Published:** 2021-05-26

**Authors:** Ayanabha Chakraborti, Christopher Graham, Sophie Chehade, Bijal Vashi, Alan Umfress, Pradeep Kurup, Benjamin Vickers, H. Alexander Chen, Rahul Telange, Taylor Berryhill, William Van Der Pol, Mickie Powell, Stephen Barnes, Casey Morrow, Daniel L. Smith, M. Shahid Mukhtar, Stephen Watts, Gregory Kennedy, James Bibb

**Affiliations:** ^1^Department of Surgery, University of Alabama at Birmingham, Birmingham, AL, United States; ^2^Department of Biology, University of Alabama at Birmingham, Birmingham, AL, United States; ^3^Department of Pharmacology, University of Alabama at Birmingham Medical Center, Birmingham, AL, United States; ^4^Center for Clinical and Translational Science, University of Alabama at Birmingham, Birmingham, AL, United States; ^5^Department of Cell, Developmental and Integrative Biology, University of Alabama at Birmingham, Birmingham, AL, United States; ^6^Department of Nutrition Sciences, University of Alabama at Birmingham, Birmingham, AL, United States

**Keywords:** diet, high fructose corn syrup (HFCS), anxiety, depression, nucleus accumbens, tryptophan, serotonin

## Abstract

The neurobiological mechanisms that mediate psychiatric comorbidities associated with metabolic disorders such as obesity, metabolic syndrome and diabetes remain obscure. High fructose corn syrup (HFCS) is widely used in beverages and is often included in food products with moderate or high fat content that have been linked to many serious health issues including diabetes and obesity. However, the impact of such foods on the brain has not been fully characterized. Here, we evaluated the effects of long-term consumption of a HFCS-Moderate Fat diet (HFCS-MFD) on behavior, neuronal signal transduction, gut microbiota, and serum metabolomic profile in mice to better understand how its consumption and resulting obesity and metabolic alterations relate to behavioral dysfunction. Mice fed HFCS-MFD for 16 weeks displayed enhanced anxiogenesis, increased behavioral despair, and impaired social interactions. Furthermore, the HFCS-MFD induced gut microbiota dysbiosis and lowered serum levels of serotonin and its tryptophan-based precursors. Importantly, the HFCS-MFD altered neuronal signaling in the ventral striatum including reduced inhibitory phosphorylation of glycogen synthase kinase 3β (GSK3β), increased expression of ΔFosB, increased Cdk5-dependent phosphorylation of DARPP-32, and reduced PKA-dependent phosphorylation of the GluR1 subunit of the AMPA receptor. These findings suggest that HFCS-MFD-induced changes in the gut microbiota and neuroactive metabolites may contribute to maladaptive alterations in ventral striatal function that underlie neurobehavioral impairment. While future studies are essential to further evaluate the interplay between these factors in obesity and metabolic syndrome-associated behavioral comorbidities, these data underscore the important role of peripheral-CNS interactions in diet-induced behavioral and brain function. This study also highlights the clinical need to address neurobehavioral comorbidities associated with obesity and metabolic syndrome.

## Introduction

Obesity is a major global health concern and in the last 50 years, its prevalence has increased worldwide, approaching pandemic levels (Blüher, 2019). Unemployment, social disadvantages and decreased socio-economic efficiency all correlate with obesity, underlining the consequent economic burden. Obesity is a major type 2 diabetes mellitus (T2DM) risk factor and a core component of metabolic syndrome (MetS). Accordingly, the increasing prevalence of obesity is paralleled by similar increases in T2DM or MetS incidence (Ginsberg and MacCallum, 2009).

As a major complication, metabolic and neuropsychiatric disorders are comorbid in humans and have been linked in several animal studies (Dunbar et al., 2008; Martins et al., 2019). Children with obesity are more prone to anxiety and depressive symptoms compared to healthy weight subjects (Ljungberg et al., 2020). The association between MetS and anxiety (Tang et al., 2017) as well as depression is well documented (Butnoriene et al., 2014). Anxiety disorders are more common in patients with T2DM than in the general population and can impact both diabetes severity and quality of life. Patients with diabetes and depressive symptoms have mortality rates nearly twice that of diabetics lacking depression symptoms (Kota et al., 2012). Despite this clear pathophysiological relationship, the molecular mechanisms that link neuropsychiatric disorders to obesity and MetS remain poorly understood.

Long-term consumption of excessive saturated fat and sugars elevates the risk of developing obesity and metabolic syndrome (Faruque et al., 2019). In the United States, HFCS is widely used as a replacement of sucrose and other sweeteners, most notably as the primary sweetener in sugar sweetened beverages, pastries, desserts, candy and various processed foods (Bray et al., 2004; Bray, 2013). A number of studies have shown that HFCS consumption in particular increases the risk for obesity and other adverse health outcomes (Moeller et al., 2009; Rippe and Angelopoulos, 2013). HFCS is a critical nutritional factor linked to increased prevalence of T2DM (Goran et al., 2013). Although the cardiometabolic effects of high levels of HFCS consumption is well-established (Pollock et al., 2012; DeChristopher et al., 2020), the neurological consequences of consumption of a diet enriched in HFCS along with moderate fat, have not been fully characterized.

The ventral striatum (nucleus accumbens, NAc) is a primary brain circuitry hub which mediates motivation for food and contributes to depression, anxiety, and goal-directed behaviors (Gruber et al., 2009). Synaptic plasticity within mesocorticolimbic circuitry is implicated in both major depressive disorder and increased susceptibility to stress (Christoffel et al., 2011). Diet induced changes in the ventral striatum have been implicated in enhanced palatable food seeking both in obese individuals as well as in rodent models of obesity (Kenny, 2011). Thus, study of how consumption a diet rich in HFCS and moderate fat affects neuronal signaling within the ventral striatum may provide critical information to better understand mental illness comorbidities associated with metabolic syndrome and obesity.

Research over the past decade has substantially improved our understanding of how the gut microbiota contributes to host physiology, behavior and a range of neuropsychiatric disorders (Kim and Shin, 2018). Gut-microbiota, by influencing the regulation of energy balance and neurotransmitter signaling, may play an important role in CNS alterations associated with metabolic disorders. Perturbations in gut microbiota may influence dopaminergic, serotonergic, GABAergic and noradrenergic, neurotransmission (Strandwitz, 2018). Dopamine system dysregulation is associated with the pathophysiology of neuropsychiatric disorders and microbiota may modulate host dopamine synthesis/catabolism (Valles-Colomer et al., 2019). Interestingly, metabolic disorders like diabetes and obesity have been associated altered dopaminergic neurotransmission (Wang et al., 2001; Pérez-Taboada et al., 2020). Levels of tryptophan, - the main serotonin (5-HT) precursor have been shown to be regulated by microbiota (O’Mahony et al., 2015). Altered serotonergic signaling in the brain is a key factor in the pathogenesis of depression and mood disorders (Marazziti, 2013). Indeed, lowering 5-HT function through the depletion of tryptophan has a mood-lowering effect in patients recovering from depression and those with seasonal affective disorder (Moore et al., 2000). Conspicuously, circulating tryptophan concentrations are decreased in people with obesity and diabetes (Favennec et al., 2015; Rebnord et al., 2017; Zapata et al., 2018).

With these considerations in mind, we formulated the HFCS-MFD as a modified version of a recently published ‘standard American diet’ (Totsch et al., 2017) and compared its long-term consumption to a defined control diet (CD) across diverse parameters including metabolic phenotype, anxio-depressive-related behavior, gut microbiota composition, serum tryptophan metabolite profile, and ventral striatal signal transduction state. Our studies indicate that HFCS-MFD-induced obesity and metabolic impairment may be linked to maladaptations in behavior and ventral striatal function.

## Materials and Methods

### Animals and Diet

4-week-old C57Bl/6J male mice were obtained from Jackson Laboratories. Mice were housed 3 animals per cage and maintained on a standard 12-hour light/12-hour dark cycle with food and water available *ad libitum.* Mice were acclimated for 1 week after which they were assigned a Control Diet (CD) (TD 94048, Envigo, Madison, WI) or a High Fructose Corn Syrup-Moderate Fat Diet (HFCS-MFD) (TD 180061) and maintained on each respective diet for 16 weeks. For CD, the source of carbohydrate included corn starch, sucrose and maltodextrin. In contrast, the HFCS-MFD contained HFCS as the primary source of carbohydrate at a level of 26% of the diet by weight. The HFCS-MFD also was comprised of 38.6% kcal from fat while the CD sourced 10.3% kcal from fat. The sole source of fat in the CD was soybean oil while the HFCS-MFD contained multiple sources of fat including palm oil, corn oil, cottonseed oil, lard, beef tallow, and anhydrous milk fat in addition to soybean oil. Moreover, HFCS-MFD contained reduced levels of fiber and increased levels of sodium and cholesterol. Animals were kept on diet for 16 weeks. Each week, individual animal body weights and food consumption per cage were measured. Caloric content of food intake was determined using the following conversions: CD = 3.6 Kcal/g; HFCS-MFD = 4.0 Kcal/g. Energy efficiency was determined by the following formula: energy efficiency = [(body weight gain/energy intake)x100] (Surwit et al., 1995). All experiments were approved by the Institutional Animal Care and Use Committee at the University of Alabama at Birmingham (UAB). All tests and measurements were carried out during the light phase of the light/dark cycle.

### Body Composition Analysis

*In vivo* body composition analysis of fat mass and lean mass was performed at baseline and following 4, 8, and 12 weeks of diet by Quantitative Magnetic Resonance (QMR) (EchoMRI^TM^ 3-in-1 composition analyzer; Echo Medical Systems, Houston, TX) (Kim et al., 2014) at the Small Animal Phenotyping Core at the University of Alabama at Birmingham.

### Glucose and Insulin Tolerance Tests

After 12 weeks of HFCS-MFD or CD, glucose tolerance testing was conducted using a handheld glucometer as previously described (Andrikopoulos et al., 2008; Benedé-Ubieto et al., 2020). Briefly, blood glucose (mg/dL) was measured from tails of fasted (4 h) mice to establish a baseline after which, mice were injected with D-glucose (2 g/kg body weight) intra-peritoneally (i.p). Blood glucose concentrations were determined at 15, 30, 60, and 120-min following D-glucose injection. Insulin tolerance tests were conducted following 12 weeks of HFCS-MFD or CD with blood glucose determined using a hand held glucometer (Benedé-Ubieto et al., 2020). Blood glucose of fasted (4 h) mice was recorded at baseline from an incision of the distal tail. Afterward fasted mice were i.p. injected with insulin (0.5 UI/kg bw). Blood samples were collected at 15, 30, 45, 90, and 120 min post insulin injection and blood glucose concentration (mg/dL) were determined.

### Behavioral Assessments

#### Open Field Test

The open field test is used for assessment of locomotor activity and anxiety like behavior (Keers et al., 2012). Briefly mice were habituated to the testing room (1 h), then placed in the same starting corner of a novel open field arena (40 cm × 40 cm × 40 cm) and allowed to freely explore for 5 min after which they were returned to their home cage. The floor of the open field apparatus was virtually divided into 16 equal squares where the four innermost squares comprised the inner zone and the twelve outer squares adjacent to the arena walls comprised the outer zone (Dey et al., 2018). The illumination was kept at 100 lux (Ueno et al., 2020). Automated recording and calculation of distance traveled, inner zone entries, and duration spent in the inner zone were determined using the Ethovision XT15 (Noldus Information Technology) video tracking software.

#### Elevated Plus-Maze Test

The elevated plus maze test is a well validated test to assess anxiety like behavior in rodents (Walf and Frye, 2007; Hill et al., 2013). For this test, mice were allowed to habituate to the testing room for 1 h and then placed in the center of a plus shaped maze raised 100 cm from the ground. The maze contained two open arms and two closed arms. All arms were 33 cm long × 5 cm wide with the closed arms having 25 cm tall walls on each side. The four arms conjoined at a central crossing region. Mice were allowed to explore the maze for a duration of 5 min. The illumination was kept at 100 lux (Ueno et al., 2020). Number of entries and duration in the open and closed arms and the distance moved during the exploration period were determined using the EthoVision XT 15 (Noldus Information Technology) video tracking system.

#### Social Interaction Test

Animal sociability was tested using the three chamber social interaction test. Briefly, mice were placed in a three chambered arena and allowed to freely explore for 10 min (habituation phase) (Umemura et al., 2017). During the sociability test session, a stranger same sex mouse designated as ‘social target’ was enclosed in an inverted wire cup and placed in a side chamber while the other side chamber contained an inverted empty wire cup (inanimate object). The test mice were placed in the central chamber and allowed to freely explore the three chambers for 10 min (Umemura et al., 2017). In this paradigm, illumination was set at 70 lux (Satoh et al., 2011). The time spent in each chamber and time spent exploring the enclosed novel mouse or the empty cup were recorded and analyzed using EthoVision XT 15 (Noldus Information Technology) video tracking software.

#### Forced Swim Test

Forced swim test (FST) is widely used to assess behavioral despair in rodents. An increase in immobility over time is associated with behavioral despair and is ameliorated by treatment with antidepressants (Poleszak et al., 2019). Consistent with previous studies (Hirani et al., 2002; Plattner et al., 2015) a two trial FST was conducted. Mice were individually placed in a glass cylinder (24 cm height, 15 cm diameter) filled with water (25°C) for 10 min (pretest) and 24 h later were re-exposed for 6 minutes. The room illumination was set to 30 lux (Lee et al., 2019). The duration of immobility was measured for the last 5 minutes of the exposure period. The mice were tracked using a horizontally mounted camera adjacent to the cylinder and EthoVision XT15 software (Noldus Information Technology) was used for analysis (Fitzgerald et al., 2019).

### Gut Microbiota Analysis

Microbiota analysis of caecal digesta were carried out at the UAB Microbiome Core facility as described previously (Kumar et al., 2014). DNA was extracted from caecal content using a commercially available kit (Zymo Research Irvine, CA, United States) following manufacturers specifications. Unique barcoded primers (Kumar et al., 2014) were utilized to amplify DNA coding for the V4 region of the 16S rRNA gene using Polymerase Chain Reaction (PCR) and isolated PCR products were purified by QIAquick Gel Extraction Kit (Qiagen, Germantown, MD). Utilizing Illumina MiSeq NextGen sequencing, PCR products were sequenced corresponding to 250 bp from the V4 region of the *16S rRNA* gene. Raw data as FASTQ files were de-multiplexed, assessed for quality control (FastQ quality control), and used for library construction. Quantitative Insight into Microbial Ecology (QIIME) was used for downstream analysis. Samples were grouped using Uclust and those harboring 97% similarity were segregated into Operational taxonomic units (OTU) and subsequently assigned to different phylogenetic levels. Alpha diversity was calculated using Chao 1, Observed Species, PD Whole Tree and Shannon indices. For analysis of beta diversity, pairwise distance matrices were generated using the Bray Curtis, unweighted and weighed UniFrac metrices and used for Principal Coordinate Analysis (PCoA) (Kumar et al., 2014). The linear discriminant analysis (LDA) effect size (LEfSe) method^1^ was used to identify the most differently abundant taxa (Segata et al., 2011; Wang et al., 2018) between the CD and HFCS-MFD groups.

### Serum Tryptophan Metabolites Analysis

Following sacrifice, serum was separated from blood samples through centrifugation using BD microtainer tubes (Becton Dickinson, USA). For tryptophan analysis, 50 μl mouse serum was combined with 25 ng/ml methanolic tryptophan-d5 internal standard (Buford et al., 2020). Serum was expelled into 500 μl of acetonitrile 1.0% formic acid contained within through Phree cartridges (Phenomenex, Torrance, CA). Following a 5-minute room temperature incubation, the mixture was drawn through the sorbent into a borosilicate collection tube. Serum samples were subsequently dried (N2 gas) and reconstituted in 100 μl 0.1% formic acid (Buford et al., 2020). Samples were compared against authentic analytical standards prepared in neat solutions. LC-MS was conducted as described (Zhu et al., 2011). HPLC gradient separation occurred on an Atlantis T3 3 μm 100 x 2.1 mm column (Waters, Milford, MA) at 40 degrees Celsius. Column waste was diverted from the mass spectrometer for the first minute of the gradient. MultiQuant 3.0.3 was used for data analysis (Buford et al., 2020). Standard curves ranged from 1 – 1,000 ng/ml over 7 points. Standard curve regressions were linear with 1/x^2^ weighting for all analytes.

### Brain Microdissection and Quantitative Immunoblotting

Mice were decapitated and brains were rapidly dissected in ice-cold PBS with 50 mM NaF as described (Plattner et al., 2015). Following brain dissection 1 mm coronal brain slices were made using a brain matrix. Punches of ventral striatal tissue from 1 mm slices were subsequently microdissected and snap-frozen in dry ice (Wang et al., 2017). Quantitative immunoblotting in ventral striatal lysates was conducted as reported (Plattner et al., 2015). Immunoblots analysis of phosphorylation state-specific antibodies were normalized to total protein signals from blots.

### Statistical Analysis

All statistical analysis was conducted using Prism 9.0 software (GraphPad Software, San Diego CA). When making direct comparisons between two groups statistical significance was determined using unpaired *t*-tests. In cases of drawing multiple comparisons and repeated measured parameters, such as longitudinal diet effects, repeated measures ANOVA analysis with Bonferroni *post hoc* test was implemented. A *p* value <0.05 was considered as statistically significant.

## Results

### HFCS-MFD Consumption Induces Metabolic Impairments

**FIGURE 1:**
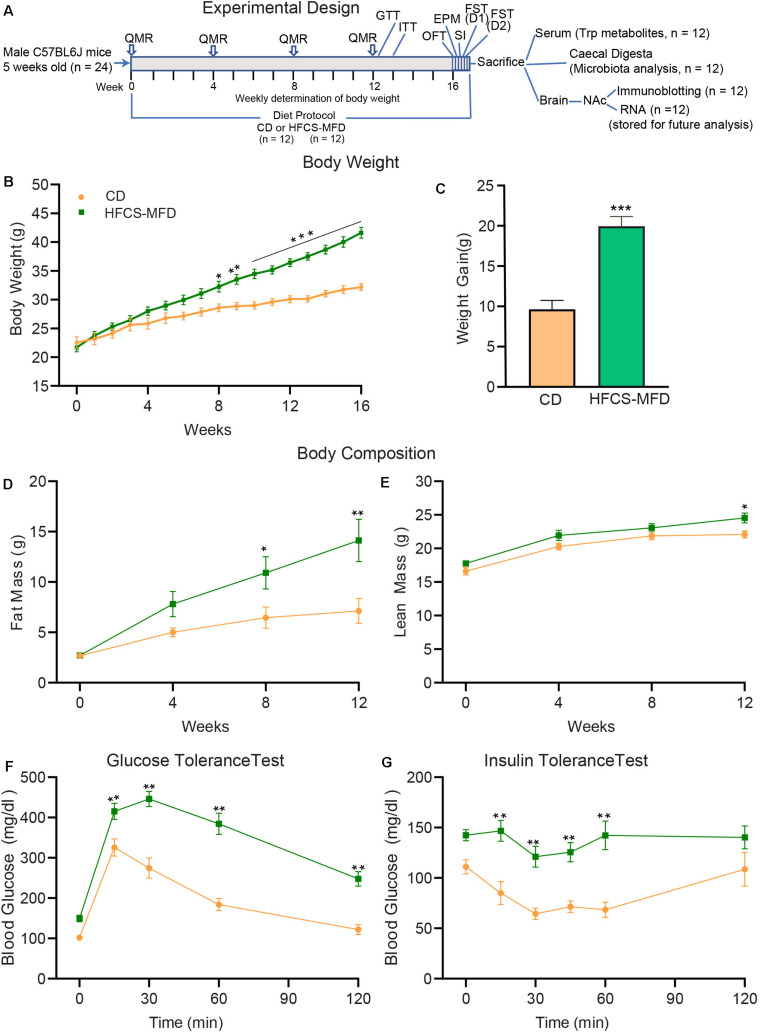
HFCS-MFD consumption induces metabolic impairments. **(A)** Schematic overview of the experimental design. Body weight, body composition analysis by QMR and metabolic parameters including glucose tolerance (GTT) and insulin tolerance (ITT) were assessed during16 weeks of CD or HFCS-MFD consumption at time points indicated. After 16 weeks of diet administration, behavioral tests including OFT, EPM, SI, FST day 1 (D1) and day 2 (D2) were conducted before samples were derived for the analyses indicated. **(B)** Body weight of CD and HFCS-MFD fed mice for 16 weeks. **(C)** Average body weight gain over 16 weeks of dietary treatments. Quantitative magnetic resonance analysis of **(D)** body fat mass and **(E)** lean body mass in CD and HFCS-MFD mice **(F)** Glucose tolerance test and **(G)** Insulin tolerance test after 12 weeks of diet. All data are Means ± S.E.M., *n* = 9-12 per group; *p < 0.05, **p < 0.01, ***p < 0.001 Repeated measures ANOVA with Bonferroni’s *post hoc* test, except for (C) which used unpaired *t*-test.

To better understand the relationship between consumption of a HFCS-MFD and altered metabolic state, following the experimental design shown in Figure 1A, 5-week-old male C57BL/6J mice were fed a HFCS-MFD or CD for 16 weeks. Body weight was recorded weekly (Figure 1B). Two-way repeated measures ANOVA showed that there were significant main effects of Diet [*F*(1,22) = 21.51, *p* < 0.0001],Time [*F*(16,352) = 177.8, *p* < 0.0001] as well as Time by Diet Interaction [*F*(16,352) = 21.10,*p* < 0.0001] on body weight following dietary exposure. *Posthoc* Bonferroni analysis revealed that HFCS-MFD mice became significantly heavier in weeks 8-16 compared to those given the CD (*p* < 0.05 at week 8; *p* < 0.01 at week 9; *p* < 0.001 at week 10-16) There was a trend for HFCS-MFD exposed mice to weigh more at the 7th week of dietary exposure (*p* = 0.06). Following 16 weeks of dietary exposure, average weight gain was significantly higher for the HFCS-MFD mice as compared to CD mice [t(22) = 6.24, *p* < 0.001] (Figure 1C). The HFCS-MFD group trended toward higher energy intake compared to the CD group (p = 0.07) (Supplementary Figure 1A). Also, energy efficiency was significantly increased in HFCS-MFD fed mice as compared to CD fed mice (*p* < 0.01) (Supplementary Figure 1B).

QMR analysis was carried out to assess body fat mass and lean body mass of the mice prior to starting diets and at weeks 4, 8, and 12 following dietary exposure. Analysis of body fat mass data showed that there were significant main effects of Diet [*F*(1,22) = 7.04, *p* < 0.01], Time [*F*(3,66) = 32.90, *p* < 0.0001] as well as Time by Diet Interaction [*F*(3,66) = 6.03, *p* < 0.001]. *Posthoc* Bonferroni analysis revealed that body fat mass was not different between the groups at baseline (*p* = 0.99) and at week 4 (*p* = 0.40). However, HFCS-MFD mice had statistically significant higher body fat mass at week 8 (*p* < 0.05) and week 12 (*p* < 0.01) compared to CD mice (Figure 1D). Analysis of lean body mass data showed that there were significant main effects of Diet [*F*(1,22) = 6.58]; Time [*F*(3,66) = 75.44] whereas Time by Diet Interaction did not attain statistical significance [(*F*(3,66) = 0.92]. *Posthoc* Bonferroni analysis revealed that body lean mass was not different between the two groups at baseline (*p* = 0.66), week 4 (*p* = 0.19) and week 8 (*p* = 0.60) while at week 12 the difference was significant (*p* < 0.05) (Figure 1E).

To further explore peripheral metabolic changes associated with the consumption of HFCS-MFD we performed glucose (Figure 1F) and insulin (Figure 1G) tolerance tests on mice after 12 weeks of diet. Analysis of glucose tolerance data showed that there were significant main effects of Diet [*F*(1,22) = 41.66, *p* < 0.0001] and Time [*F*(4,88) = 133.7, *p* < 0.0001] as well as Time by Diet Interaction [*F*(4,88) = 10.90, *p* < 0.0001] on blood glucose levels. *Posthoc* Bonferroni analysis revealed that HFCS-MFD blood glucose levels were significantly higher at 15 min (p < 0.01) as well as at 30, 60, and 120 min (*p* < 0.01 at each time point) following intraperitoneal glucose injection in the HFCS-MFD group compared to the CD group. Thus, HFCS-MFD consumption caused glucose intolerance. Similarly, analysis of insulin tolerance data showed that there were significant main effects of Diet [*F*(1,16) = 26.07, *p* < 0.0001] and Time [*F*(5,80) = 6.15, *p* < 0.0001]. While Time by Diet Interaction [*F*(5,80) = 2.29, *p* = 0.053] did not attain statistical significance a trend toward significance was apparent. *Posthoc* Bonferroni analysis revealed that there were significant differences in blood glucose levels at 15, 30, 45 and 60 min (*p* < 0.01) at each time point after intraperitoneal insulin injection in the HFCS-MFD group compared to the CD mice. These data demonstrate that the HFCS-MFD mice displayed insulin resistance. Together these results show that consumption of a HFCS-MFD is associated with profound alterations in mouse body weight, body composition and metabolic state.

### HFCS-MFD Feeding Potentiates Anxio-Depressive Behavior

**FIGURE 2:**
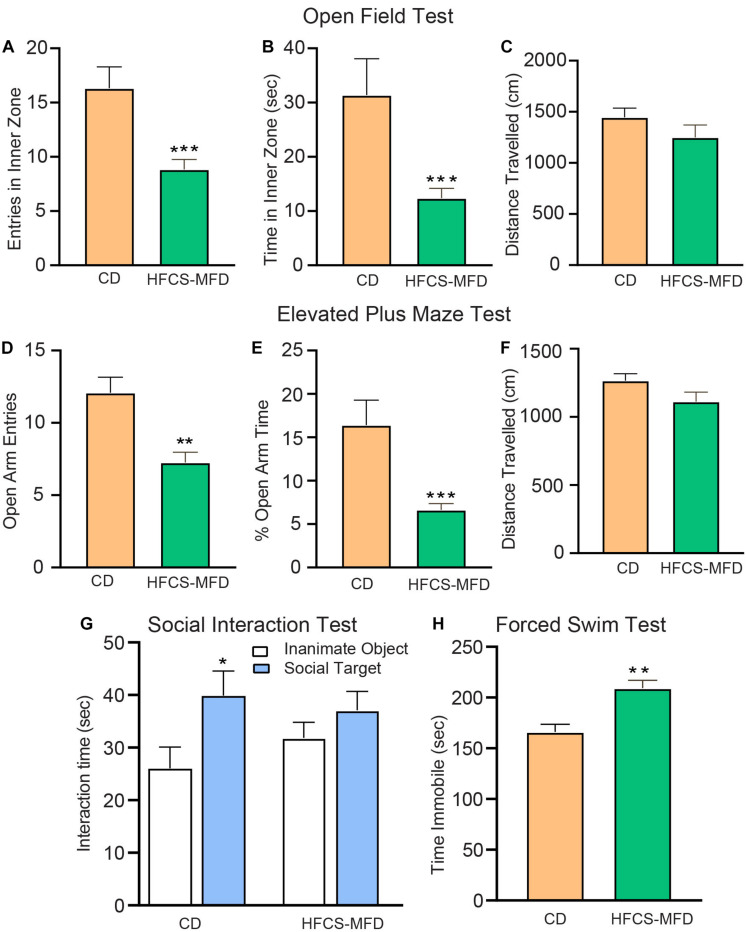
HFCS-MFD consumption potentiates anxio-depressive behavior. The effects of HFCS-MFD vs CD on **(A)** Entries in the inner zone **(B)** Time spent in the inner zone and **(C)** Distance travelled for 5 min exploration in the Open field test. **(D)** Number of open arm entries **(E)** Percent Time spent in the open arms and **(F)** Distance travelled for 5 min exploration in the elevated plus maze test **(G)** Interaction time with a social target (stranger mice) versus an inanimate object (empty cylinder) during 10 min exploration in the three chambered social interaction test **(H)** Immobility time in the last 5 min of Forced swim test (*n* = 10-12 per group; *p < 0.05 **p < 0.01 ***p < 0.001 unpaired *t*-test).

To understand how the HFCS-MFD affected brain function, behavioral assessments were carried out following 16 weeks of HFCS-MFD consumption. Anxiety-like behavior was assessed using the open field test (OFT) and elevated plus maze (EPM). The OFT is based on the aversion of rodents to novel, brightly lit, open environments (Keers et al., 2012). Interestingly, mice on the HFCS-MFD displayed a significant decrease in the duration of exploration time in the inner zone [t(22) = 3.29, *p* < 0.001] and fewer number of entries in the inner zone [t(22) = 3.73, *p* < 0.001] of the open field, as compared with the control group during the 5 min exploration period (Figures 2A,B), indicating enhanced anxiogenesis in HFCS-MFD mice. No significant difference in the total distance travelled [t(22) = 1.76, *p* = 0.09] was observed between the 2 groups (Figure 2C) indicating that HFCS-MFD consumption was not associated with locomotor deficits.

The anxiety-like effects of the HFCS-MFD were further assessed using the EPM. This test is based on the conflict between the innate tendencies of rodents to explore novel environments and avoid open and brightly lit areas (Walf and Frye, 2007). Similar to the effects observed in the OFT, HFCS-MFD mice made fewer entries [t(22) = 2.74, *p* < 0.01] and spent less % time in the open arms [t(22) = 3.47, *p* < 0.001] of the EPM (Figures 2D,E). There was however no difference in the total distance travelled [t(22) = 1.28, *p* = 0.21] (Figure 2F) and total number of closed arm entries (data not shown) between CD and HFCS-MFD mice during the 5 min exploration period.

Deficits in social interaction is a core symptom of multiple neuropsychiatric disorders (Rincón-Cortés and Sullivan, 2016). To determine the effects of the HFCS-MFD on social interaction behavior, the 3-chamber social interaction test was used (Figure 2G). CD mice spent significantly more time interacting with the social target (stranger mice) compared to time spent exploring an inanimate object (empty cylinder) [t(18 = 2.24; *p* < 0.05]. In contrast, HFCS-MFD mice did not show a preference for social interaction [t(10) = 1.12, *p* = 0.27]. Thus, HFCS-MFD consumption was associated with deficits in social interaction.

The Forced Swim Test (FST) is a rodent behavioral test used to evaluate “depressive-like” states and behavioral despair in rodents (Poleszak et al., 2019). Measurement of the duration of immobility when rodents are exposed to an inescapable situation is used as an index of behavioral despair in this test. Mice fed the HFCS-MFD exhibited significantly elevated time immobile compared with CD-fed mice [t(19) = 4.03, p < 0.01] suggesting an increased disposition toward behavioral despair (Figure 2H). Collectively, these results demonstrate increased anxiety and depressive-like behavior resulting from long-term consumption the HFCS-MFD.

### HFCS-MFD Consumption Alters Gut Microbiota Composition

**FIGURE 3:**
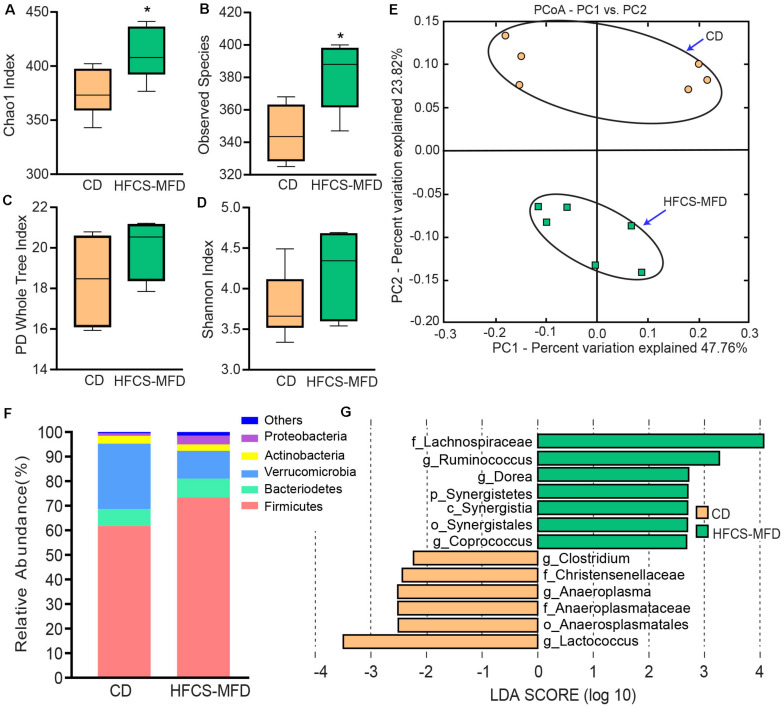
HFCS-MFD consumption alters gut microbiota composition. Box plots showing differences in the caecal microbiome alpha diversity indices between the CD and HFCS-MFD groups according to the **(A)** Chao1 **(B)**, Observed Species **(C)** PD Whole Tree and **(D)** Shannon diversity indices based on OTU levels. (*n* = 6 per group; *p < 0.05 Wilcoxon Rank Sum test). **(E)** Principal Coordinate Analysis (PCoA) plot based on unweighted UniFrac distance between samples **(F)** Relative abundances of the bacterial communities at the phylum taxonomic rank among the CD and HFCS-MFD mice (*n* = 6 per group) **(G)** Linear discriminant analysis (LDA) effect size (LEfSe) analysis of gut microbiota changes following consumption of 16 weeks of HFCS-MFD or CD (*p* < 0.05; LDA > 2). The colors represent the group in which the indicated taxa are more abundant compared to the other group.

Alterations in gut microbiota are increasingly implicated in the pathophysiology of neurological and neuropsychiatric disorders. To explore the relationship between HFCS-MFD-induced changes in behavioral phenotype and the gut microbiome, contents of the caecum (digesta) were collected and processed for microbiota analysis following behavioral testing. Alpha diversity analysis showed that Chao 1 and Observed Species indices (denoting species richness) were significantly increased (*p* < 0.05, Wilcoxon rank sum test) in the HFCS-MFD group (Figures 3A,B). On the other hand, phylogenetic diversity (represented by PD whole tree), and richness and evenness (represented by Shannon index) of the microbial community was not significantly different between HFCS-MFD and CD mice (Figures 3C,D).

Assessment of the beta diversity revealed statistically significant dissimilarities (PERMANOVA analysis) between the CD and HFCS-MFD in all metrices including Bray–Curtis (p = 0.005) unweighted UniFrac (*p* = 0.025) and weighted UniFrac (*p* = 0.042). Consequently, microbial communities clustered differently in the CD and HFCS-MFD groups in Principal coordinate analysis plot based on unweighted UniFrac distance (Figure 3E).

Analysis of the relative abundances of the bacterial populations at the phylum level revealed trends toward higher abundance in Firmicutes (*p* = 0.06) and decreased abundance for Verrucomicrobia (*p* = 0.06) (Figure 3F). The differences in the relative abundances of Bacteroidetes, Actinobacteria and Proteobacteria were not statistically distinguishable between CD and HFCS-MFD mice.

To further resolve differences between the two groups a linear discriminant analysis (LDA) effect size (LEfSe) algorithm was used to identify more abundant taxa in one group compared to the other (Figure 3G). LEfSe analysis revealed thirteen discriminative features at different taxonomic levels. Bacteria belonging to class *Synergistia*, order *Synergistales*, family *Lachnospiraceae*, and genera *Ruminococcus, Coprococcus, and Dorea* were more abundant in the HFCS-MFD group whereas bacteria belonging to families *Anaeroplasmataceae* and *Christensenellaceae*, order *Anaeroplasmatales*, and genera *Anaeroplasma*, *Lactococcus* and *Clostridium* were more abundant in the CD group. These data demonstrate that a shift occurs in the gastrointestinal microbiota as a result of HFCS-MFD consumption.

### HFCS-MFD Consumption Dysregulates Serum Tryptophan Metabolism

**FIGURE 4:**
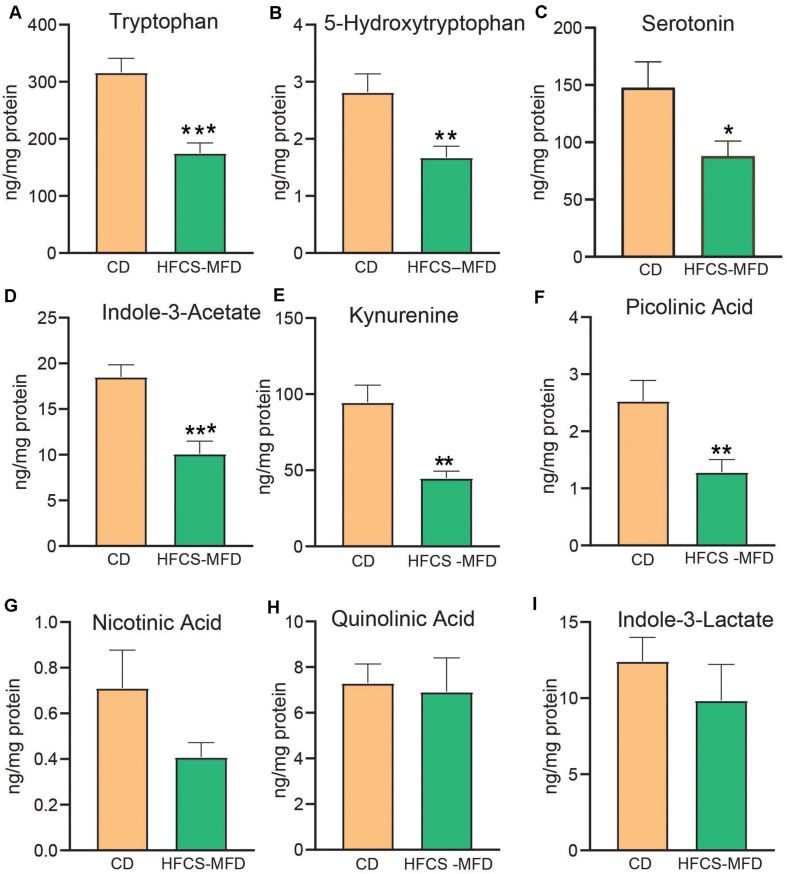
HFCS-MFD dysregulates serum tryptophan metabolism. The effects of the HFCS-MFD vs CD on serum levels of **(A)** tryptophan, **(B)** 5-hydroxytryptophan, **(C)** Serotonin, **(D)** Indole-3-Acetate, **(E)** Kynurenine, **(F)** Picolinic Acid, **(G)** Nicotinic Acid, **(H)**, Quinolinic acid and **(I)** Indole-3-lactate following 16 weeks of diet are shown, (*n* = 6 per group; *p < 0.05, **p < 0.01, ***p < 0.001 unpaired *t*-test).

Circulating and brain levels of tryptophan and its metabolites have been implicated as factors in depression, schizophrenia, and other psychiatric disorders (Hughes et al., 2012). Tryptophan is the precursor of serotonin, and quantitative assessment of its metabolites can assist in understanding the pathophysiological mechanisms of psychiatric disorders and evaluating corresponding therapeutic interventions. Here, liquid chromatography with tandem mass spectrometry (LC-MS/MS) was used to assess the effects of HFCS-MFD consumption on serum tryptophan metabolite levels. HFCS-MFD significantly lowered serum levels of tryptophan [t(10) = 4.69, *p* < 0.001] and its immediate metabolite, 5-hydroxytryptophan (5-HTP) [t(10) = 3.04, *p* < 0.01] (Figures 4A,B). The level of the neurotransmitter serotonin was also lower in the HFCS-MFD group [t(10) = 2.29, *p* < 0.05] (Figure 4C). Other tryptophan metabolites that were significantly reduced in HFCS-MFD included indole-3 acetate [t(10) = 4.43; p < 0.001] (Figure 4D), kynurenine [t(10) = 4.10, *P* < 0.01] (Figure 4E), and picolinic acid [t(10) = 2.96, *p* < 0.01] (Figure 4F). In contrast, the serum levels of nicotinic acid [t(10) = 1.71, *p* = 0.11] (Figure 4G) quinolinic acid [t(10) = 0.22, *p* = 0.82] (Figure 4H) and indole-3-lactate[t(10) = 0.90, *p* = 0.38] (Figure 4I) were not significantly different between the two groups. These findings demonstrate that HFCD consumption attenuates the serum levels of the neuroactive amino acid, tryptophan and several of its direct metabolites.

### HFCS-MFD Consumption Alters Ventral Striatal Neuronal Signaling

**FIGURE 5:**
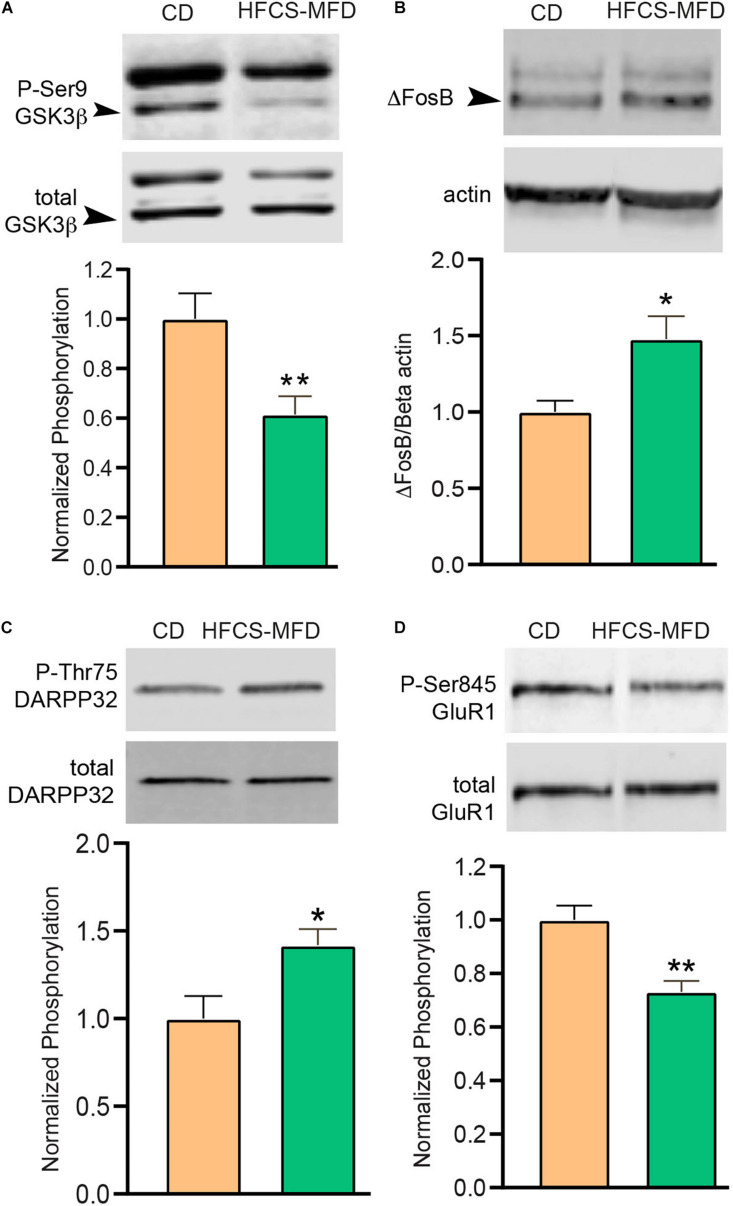
HFCS-MFD alters ventral striatal neuronal signaling. The effects of HFCS-MFD vs CD on **(A)** phospho-Ser9GSK3β, **(B)** ΔFosB, **(C)** phospho-Thr75 DARPP-32 and **(D)** phospho-Ser845GluR1 protein levels in ventral striatal lysates of mice following 16 weeks of diet are shown, (*n* = 4-6 per group; *p < 0.05, **p < 0.01 unpaired *t-*test).

Depression, anhedonia and anxiety disorders have been associated with structural and functional changes in the ventral striatum (nucleus accumbens, NAc) (Heshmati and Russo, 2015). Ventral striatal function is dictated by cellular processes including neurotransmission, signal transduction, and gene expression. The protein kinase GSK3β has been implicated in mediating anxio-depressive disorders (Jope and Roh, 2006). The transcriptional factor ΔFosB mediates both reward and aversive behaviors in the ventral stratum (Wallace et al., 2008). The signaling integrator DARPP-32 coordinates dopamine and excitatory neurotransmission and can be converted to a PKA inhibitor via Cdk5-dependent phosphorylation of Thr75 (Bibb et al., 1999). AMPA receptors mediate excitatory neurotransmission and phosphorylation of Ser845 of the GluR1 subunit of AMPA receptors is a determinant of synaptic cell surface availability (Oh et al., 2006). To determine if HFCS-MFD-induced behavioral and metabolomic changes affected each of these signaling pathways, ventral striatal lysates were subjected to quantitative immunoblotting. While overall expression of the total GSK3β were similar in both groups, inhibitory phosphorylation of Ser 9 GSK3β was significantly reduced [t(10) = 3.02, *p* < 0.01] in HFCS-MFD mice (Figure 5A) suggesting that HFSCD led to increased activation of GSK3β in this brain region. The levels of the transcription factor ΔFosB, which is upregulated during chronic stress (Perrotti et al., 2004), were significantly increased in NAc lysates from HFCS-MFD-fed mice [t(7) = 2.63, *p* < 0.05] (Figure 5B). HFCS-MFD also increased phosphorylation of Thr75 DARPP-32 [t(6) = 2.64, *p* < 0.05] (Figure 5C) suggesting Cdk5 activation and attenuation of PKA activity and dopamine neurotransmission. The phosphorylation state of Ser845 GluR1 was decreased [t(9) = 4.04, *p* < 0.01] (Figure 5D) also consistent with reduced PKA activity and possible alterations in ventral striatal medium spiny neuron excitability. Together, these data show that consumption of a HFCS-MFD alters signaling pathways that mediate ventral striatal function and anxiety and depression related behavior.

## Discussion

The pandemic of obesity, metabolic syndrome and diabetes represents a major public health concern (Ginsberg and MacCallum, 2009; Blüher, 2019). HFCS and fat are ubiquitous in Western style diets and likely contribute to increased prevalence of obesity (Bray et al., 2004; Bocarsly et al., 2010). Neuropsychiatric comorbidities associated with these conditions not only affect the quality of life of patients suffering from these conditions, but also serve as major risk factor for disease severity (Nousen et al., 2013; Castanon et al., 2014). Unhealthy dietary habits are increasingly recognized to affect brain function and neuropsychiatric and neurological disorder progression (Ljungberg et al., 2020; Popa-Wagner et al., 2020). The underlying mechanistic links between diet-induced obesity, metabolic syndrome and vulnerability to neuropsychiatric complications are not well understood. Here we showed that consumption of a HFCS-MFD potentiates anxio-depressive behavior, alters gut microbiota profile, dysregulates serum tryptophan metabolism and alters the phosphorylation state of key neuronal proteins in the ventral striatum that regulate mood and anxiety.

Most of the studies assessing effects of HFCS consumption in animal models have used HFCS in drinking water. However, HFCS-sweetened water may enhance drinking palatability and encourage replacement of solid food-derived calories with those available from fluid intake (Pan et al., 2018). Accordingly, we formulated the HFCS-MFD that incorporates HFCS within the feed and is a modified version of a recently published standard American diet (Totsch et al., 2017). In our study, HFCS-MFD fed mice gained more weight and exhibited increased fat mass when compared to mice fed the CD. These findings are consistent with an earlier report where consumption of fructose but not sucrose sweetened beverages selectively enhanced adipogenesis in mice (Jürgens et al., 2005). In contrast, mice given 10% HFCS solution in combination with *ad libitum* standard chow for 15 weeks did not undergo weight gain but did exhibit glucose dysregulation (Meyers et al., 2017). In addition to high fructose content, the diet we formulated was comprised of elevated saturated fat as well as omega-6 PUFA, which have been associated with an increased risk of obesity. These factors may also have contributed to the hyperglycemia and reduced insulin sensitivity phenotype we observed. Rats given HFCS solution showed similar glucose intolerance (Alten et al., 2018) and HFCS intake have been reported to induce insulin resistance in both rodents and non-human primates (Bremer et al., 2011). Thus, the current study is consistent with others where HFCS intake caused similar metabolic outcomes.

Our findings showed that mice exposed to long-term HFCS-MFD exhibited anxiety-like behavior in both the open field and elevated plus maze, two widely validated tests to assess anxiety in rodents. Similarly, previous studies have shown that rats administered 65% fructose in drinking water induce anxiogenic-like behaviors (Reddy et al., 2016). On the other hand, rats given *ad libitum* access to a 11% HFCS solution for 4 weeks during adolescence and behaviorally tested during adulthood did not retain enhanced anxiety although some cognitive deficits persisted (Noble et al., 2019), suggesting that systemic recuperative plasticity may have limitations.

HFCS-MFD mice also exhibited behavioral despair as evidenced by increased immobility in the FST. Likewise, adolescent rats administered 11% HFCS solution exhibited similar maladaptive behavior (Alten et al., 2018). While increased adiposity may impair swimming ability, no difference was detected in general locomotor activity between the CD and HFCS-MFD group, suggesting the effect was mood related. In addition to despair, social behavior deficits are a hallmark of psychiatric disorders, including depression, anxiety, and schizophrenia (Rincón-Cortés and Sullivan, 2016). Reduced social interactions observed in HFCS-MFD-fed mice is consistent with earlier findings that high fat diet induces a depression-like phenotype with reduced sociability and anhedonia in mice (Hassan et al., 2019). Overall, these results add to a growing body of preclinical and clinical findings that suggest deleterious effects of high fat and/or fructose diet on neurobehavior.

An imbalance of the intestinal microbiota may contribute to a variety of disorders such as obesity or type 2 diabetes, and can also play a role in the development of psychiatric disorders (Huang et al., 2019). Diet, antibiotics and variety of environmental factors can modify gut microbiota composition (Leeming et al., 2019). Our findings show that HFCS-MFD mice had an increased abundance of bacteria belonging to genus *Ruminococcus*. In accordance case-control studies have reported that patients with generalized anxiety disorder have overgrowth of *Ruminococcus gnavus* compared to healthy controls (Jiang et al., 2018). Bacteria belonging to family *Lachnospiraceae* have been associated with social avoidance behaviors in mice (Schnorr and Bachner, 2016) and is consistent with our findings of increased abundance of *Lachnospiraceae* as well as impaired social interaction in HFCS-MFD mice. We also found that HFCS-MFD consumption led to decreased abundance of bacteria belonging to the genus *Clostridium*. These bacteria are commonly altered in conjunction with effects on the production of intestinal metabolite such as phenylalanine, tryptophan, and tyrosine which may affect gut–brain axis signaling. *Clostridium* metabolizes tryptophan into indole and subsequently 3-indolepropionic acid (IPA) (Wikoff et al., 2009), which confers neuroprotective effects via its antioxidant properties (Zhang and Davies, 2016). Furthermore, abundance of *Lactococcus* was also reduced in HFCS-MFD mice. Bacteria belonging to genus *Lactococcus* are used in probiotics and have been suggested to have anxiolytic effects (Surzenko et al., 2020). Thus, several of the effects of HFCS-MFD consumption are consistent with the growing link between the gastrointestinal microbiome and neuropsychiatric/neurological health.

Serotonergic transmission is critical to normal neurological function and lower serotonin (5-HT) levels can provoke diverse pathophysiological abnormalities, most of which are reflected in dysfunctional behavioral output (Sandyk, 1992). 5-HT is synthesized from essential amino acid precursor L-tryptophan. The bioavailability of tryptophan is the principal rate-limiting factor for 5-HT synthesis. Mice fed HFCS-MFD showed reduced serum levels of tryptophan as well as 5 hydroxytryptophan (the immediate precursor of serotonin), both of which can cross the blood brain barrier. Therefore, reduced availability of its precursors can lead to lower brain serotonin levels and in turn may contribute to the behavioral impairments observed in HFCS-MFD mice. Interestingly, the HFCS-MFD also reduced picolinic acid, an endogenous metabolite of L-tryptophan, which may have a wide range of neuroprotective effects (Grant et al., 2009). HFCS-MFD induced alterations in serum tryptophan metabolites levels may therefore be an important communication link by which diet-induced changes in the gastrointestinal system imparts effects upon brain function.

In addition to serotonin, other monoamine neurotransmitters including dopamine and norepinephrine also play important roles in mood regulation (Lanni et al., 2009). Reciprocal relationships exist between populations of monoamine, serotonin (5-HT), norepinephrine (NE) and dopamine (DA) neurons in the brain (Guiard et al., 2008). Dopamine-D2 receptor neurotransmission is altered in the brains of obese animal models and humans (Wang et al., 2001; Fetissov et al., 2002; Geiger et al., 2009). Dopamine function may also be altered in patients with diabetes as well as in rodents consuming a high fructose diet (Pijl and Edo, 2002; Meyers et al., 2017). Sucrose consumption decreases tyrosine hydroxylase (a catecholamine metabolizing enzyme), in the prefrontal cortex and alters dopamine signaling in rats (Tobiansky et al., 2020). Further studies of how our HFCS-MFD alter metabotropic monoamine, as well as ionotropic excitatory neurotransmission, are warranted so that a more direct link between the diet and brain circuitry function can be established.

Ventral striatum is a part of the cortico-limbic circuit and intracellular kinase signaling cascades serve as important mediators of ventral striatal function (Crofton et al., 2017). GSK3β is a multifunctional serine/threonine protein kinase involved in the modulation of various aspects of neuronal function and dysregulation of this kinase has been implicated in mood disorders (Jope and Roh, 2006). We showed that that Ser9 GSK-3β phosphorylation was significantly reduced in the ventral striatum of HFCS-MFD mice, indicating increased activation of this kinase. GSK3β have been reported to be activated in the brains of mice exhibiting depression like behavior (Polter and Li, 2011), as well as in the NAc of mice subjected to social defeat stress (Wilkinson et al., 2011). GSK3β activity is regulated by serotonergic neurotransmission. Stimulation of 5-HT1 and 5-HT7 increases phospho-Ser9 GSK3β, while activation of 5-HT2AR has the opposite effect (Duda et al., 2020). Pharmacological activation of 5-HT signaling through acute administration of d-fenfluramine increases phospho-Ser 9 GSK3β in cerebral cortex, hippocampus and striatum (Li and Jope, 2010). Inhibition of GSK3β via phosphorylation of Ser 9 may also serve as an important regulatory step in the antidepressant effect of fluoxetine (Polter and Li, 2011). It is therefore tempting to speculate that, HFCS-MFD-induced peripheral tryptophan reduction may lower ventral striatal serotonergic signaling which activates GSK3β that in turn contributes to the anxio-depressive behavior observed in these mice. Assessment of ventral striatal serotonin levels and evaluating the activation state of serotonin receptors in this brain region following HFCS-MFD consumption will be helpful in further delineating the involvement of this signaling mechanism in HFCS-MFD-associated neurobehavioral alterations.

Besides, activation of GSK3β, HFCS-MFD also increased ventral striatal ΔFosB expression. Prolonged induction of ΔFosB occurs within NAc and other reward related brain regions in response to chronic stress and may mediate the long-term effects of stress on the brain (Perrotti et al., 2004; Nestler, 2015). Disruption of homeostatic integration of neurotransmitter and neuromodulator signals within the striatum underlies the pathophysiology of many neuropsychiatric disorders. DARPP-32, a neuronal phosphoprotein integrates dopaminergic and glutamatergic inputs to striatal medium spiny neurons (Fernandez et al., 2006). Under dopaminergic stimulation, DARPP-32 is phosphorylated by cAMP-dependent Protein Kinase A (PKA) at Thr34 thus inhibiting protein phosphatase-1(PP1). In contrast when DARPP-32 is phosphorylated at Thr75 by Cdk5 it becomes an inhibitor of PKA (Bibb et al., 1999; Engmann et al., 2015). HFCS-MFD increased phosphorylation of Thr75 DARPP-32, suggesting Cdk5 activation and attenuation of PKA activity. Accordingly, increased Thr75 DARPP-32 phosphorylation status in the NAc has recently been shown to contribute to comorbid depressive-like behaviors in a preclinical model of Huntington’s disease (Brito et al., 2019). Furthermore, the phosphorylation state of Ser845 GluR1 was decreased in ventral striatal lysates consistent with reduced PKA activity and suggesting that NAc synaptic excitability may be affected by HFCS-MFD consumption. The effects of HFCS-MFD on ventral striatal signaling provides an intriguing mechanism of how it may enact its deleterious effects on neurobehavior. These results also illustrate the need for more detailed study of the effects of obesity and diabetes on mood-related brain circuitry so that new mechanisms may be discovered for future targeted interventions.

Recent studies have highlighted role of insulin and insulin receptors in depression and other mood disorders (Zou et al., 2020). The insulin signaling pathway not only mediates metabolic homeostasis, but also plays an important role in, neuronal survival, dendritic arborization and synaptic plasticity thereby serving to link metabolic function and mood state (Banks et al., 2012; Lee et al., 2016). Insulin receptors are expressed in different brain regions involved in mood regulation including hypothalamus, hippocampus and nucleus accumbens (Lee et al., 2016). Insulin receptor knockdown in the hypothalamus triggered anxiety and depressive like behaviors in rodent models (Grillo et al., 2011). Insulin may influence excitatory transmission via both pre-synaptic and post-synaptic mechanisms in the NAc (Fetterly et al., 2021). Insulin signaling has also been reported to alter serotonergic neurotransmission (Martin et al., 2021). Moreover, high fat diets alter insulin signaling in brain regions including the amygdala and hypothalamus (Oh et al., 2013). Based on our findings, that HFCS-MFD consumption induces insulin resistance it would be interesting to explore in subsequent studies the effects of HFCS-MFD on insulin receptor signaling within the limbic system and how such changes may potentially relate to neurobehavioral dysfunction.

The goal this study was to compare the effects of a diet that mimics junk food with high levels of fructose and appreciable fat content to a normal chow diet. Given the array of effects, a next step would be to determine the degree to which HFCS vs fat, or any of the other variables between these two diets combined contributes to the observed effects. It will also be important to incorporate female subjects in future studies to assess any sex specific susceptibility to HFCS-MFD intake. Sex differences in the effects of high calorie diet consumption has previously been reported but the findings are not consistent. For example, Hwang et al. (2010) found that male mice are more vulnerable than females to the impacts of a high fat diet on changes in metabolic parameters and alterations in cognitive function and hippocampal synaptic plasticity. In contrast, a different study showed that chronic administration of a high fat diet produced impairment of hippocampal neurogenesis in females but not in the males (Robison et al., 2020). Similarly, rats fed a high fructose diet had increased synaptic respiration in females while males showed a decrease (Kloster et al., 2021). Thus, future studies that incorporates both sex and age as biological variables are warranted.

## Conclusion

Given the growing prevalence of metabolic syndrome, obesity, and neuropsychiatric disorders worldwide, unraveling the complex mechanisms by which unhealthy dietary factors can perturb emotional and motivational processes is urgently needed. The results presented here show important intersystemic interactions between brain function and metabolic state, underling the need to better understand this interaction and to develop interventions that are more effective at disrupting motivational drive to consume unhealthy diets.

## Data Availability Statement

The datasets presented in this study can be found in online repositories. The names of the repository/repositories and accession number(s) can be found below: NCBI BioProject repository with Accession ID: PRJNA718626.

## Ethics Statement

The animal study was reviewed and approved by Institutional Animal Care and Use Committee, University of Alabama at Birmingham.

## Author Contributions

AC, CG, SC, BV, AU, PK, BV, HC, RT, TB, and WV conducted the experiments and analyzed data. AC, CG, MP, SB, CM, DS, MM, SW, GK, and JB designed the experiments and directed this research. AC, CG, MM, SW, GK, and JB wrote the manuscript. All authors approved the manuscript.

## Conflict of Interest

The authors declare that the research was conducted in the absence of any commercial or financial relationships that could be construed as a potential conflict of interest.

## Footnotes

1 http://huttenhower.sph.harvard.edu/lefse/
