# Supplementary material for: High Fructose Corn Syrup-Moderate Fat Diet Potentiates Anxio-Depressive Behavior and Alters Ventral Striatal Neuronal Signaling
Source: Front Neurosci. 2021 May 26;15:669410. doi: 10.3389/fnins.2021.669410 (PMC8187874; doi:10.3389/fnins.2021.669410)

SUPPLEMENTARY FIGURE

SUPPLEMENTARY FIGURE 1. HFCS-MFD exhibits higher energy efficiency than CD. Quantitation of calorie intake and energy efficiency for the two diets are shown. All data are means ± S.E.M.; **p < 0.01, unpaired *t*-test.

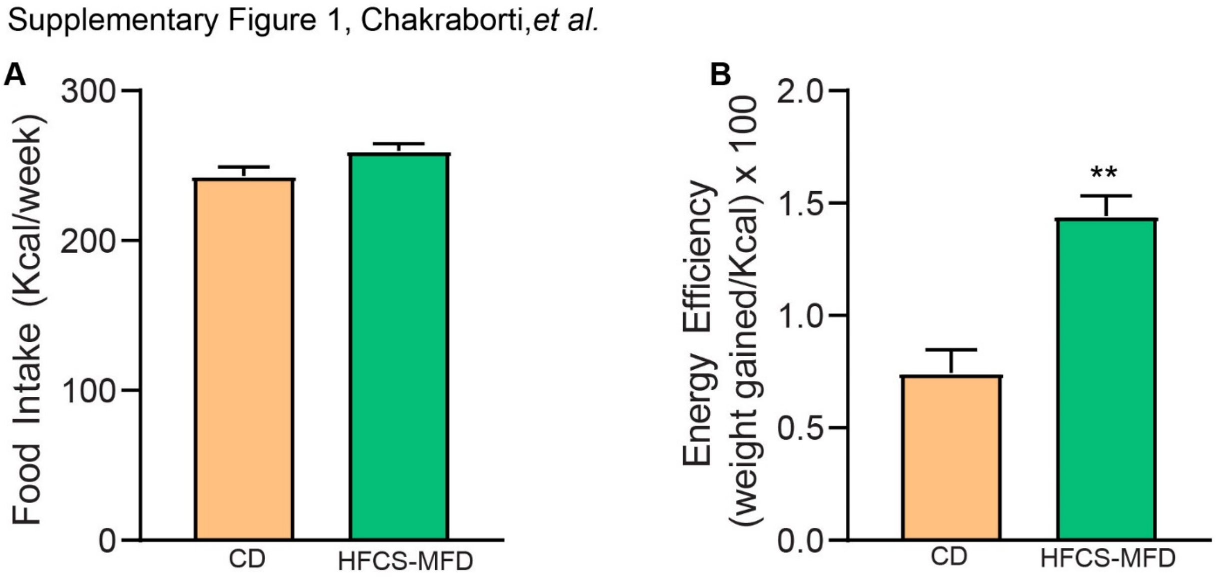

Supplement: Supplementary file 1 [file Image_1.pdf]
